# Supplementary material for: Activation and Evasion of the FEAR Pathway by RNA Viruses
Source: bioRxiv. 2025 Feb 25:2024.08.22.609092. Preprint. [Version 2] doi: 10.1101/2024.08.22.609092 (PMC11888236; doi:10.1101/2024.08.22.609092)
Supplement: Supplement 1 [file NIHPP2024.08.22.609092v2-supplement-1.pdf]

bioRxiv preprint doi: https://doi.org/10.1101/2024.08.22.609092; this version posted February 25, 2025. The copyright holder for this preprint (which was not certified by peer review) is the author/funder, who has granted bioRxiv a license to display the preprint in perpetuity. It is made available under aCC-BY 4.0 International license.

Supplementary Figure Legends

bioRxiv preprint doi: https://doi.org/10.1101/2024.08.22.609092; this version posted February 25, 2025. The copyright holder for this preprint (which was not certified by peer review) is the author/funder, who has granted bioRxiv a license to display the preprint in perpetuity. It is made available under aCC-BY 4.0 International license.

Fig S1. VSV-induced ETS-1 expression is independent of RIG-I or IFN signaling. (A, B) Representative IB (A) and quantification (B) of ETS-1 expression using IB of WCE from control or RIG-I knockout (KO) A549 cells infected with the indicated strains (MOI=10) for 8 h. Note: RIG-I is expressed at a low level in uninfected cells and is known to be induced by viral infection [73, 74], consistent with our results. (C, D) Representative IB (C) and quantification (D) of ETS-1 expression using IB of WCE from control or IRF3 KO A549 cells infected with the indicated strains (MOI=10) for 8 h. (E, F) Representative IB (E) and quantification (F) of ETS-1 expression using IB of WCE from control or STAT1 KO A549 cells infected with the indicated strains (MOI=10) for 8 h. Data in B, D, and F are means ± SD for n=3 experiments. Statistical significance in B, E, and F were determined by unpaired two-tailed Student's t-test between indicated treatments. For brevity, only the most relevant statistical comparisons are shown. * = P< 0.05; ** = P< 0.01; ns, not significant.

Fig S2. The VSV^{M51R}-eGFP strain is unable to deplete hSpt16^{SUMO} regardless of MOI. IB of endogenous hSpt16 in A549 WCE after infection with VSV-eGFP or VSV^{M51R}-eGFP at the indicated MOI. GFP is used as a marker for infection.

Fig S3. A VSV$^{\Delta M51}$ strain is unable to deplete hSpt16^{SUMO}. IB of endogenous hSpt16 in A549 WCE after infection with VSV-GFP (WT) or VSV$^{\Delta M51}$-GFP (ΔM51) (MOI=3). GFP is used as a marker for infection.

Fig S4. VSV M-mediated depletion of hSpt16^{SUMO} is independent of Rae1 and Nup98. IB of endogenous hSpt16 in A549 whole cell extract (WCE) 72 h after RNAi of

bioRxiv preprint doi: https://doi.org/10.1101/2024.08.22.609092; this version posted February 25, 2025. The copyright holder for this preprint (which was not certified by peer review) is the author/funder, who has granted bioRxiv a license to display the preprint in perpetuity. It is made available under aCC-BY 4.0 International license.

Rae1 (A) or Nup98 (B) under mock- or VSV-eGFP-infection (MOI = 10) conditions for 12 h. VSV N and GFP are markers for infection. Scram., scrambled.

Fig S5. Human and *Lymantria dispar*-encoded Spt16 proteins share a similar A51R-binding domain. Alignment of the VV A51R-binding domain of hSpt16 [6] (accession: NP_009123.1) with corresponding region in LdSpt16 (accession: GCA_004115105.1). The overall amino acid identity of these regions is 72.8%. Alignment was made using NCBI Blast2p Software.

Fig S6. SeV C' promotes depletion of SUMOylated Spt16 subunits present in the cytosol. IB of HA-hSpt16 or HA-hSpt16$^{\Delta NLS}$ in 293T WCE 24 h after transfection with empty vector (EV) and or SeV C'-Flag expression constructs.

Fig S7. SeV C' promotes depletion of SUMOylated Spt16 subunits in mouse 3T3 cells. IB of endogenous mouse Spt16 in 3T3 WCE 24 h after transfection with empty vector (EV) and or SeV C'-Flag expression constructs.

Fig S8. VSV M protein structural modeling. (A) VSV M protein map showing region surrounding M51 residue (red). NTE = N-terminal extension. Protein map based on reference [58]. B) 4OWR structure of VSV M fragment (a.a. 44-229) bound to Rae1-Nup98 complex [58]. (C) Alphafold2-generated structure of full-length (a.a. 1-229) VSV M (strain Indiana). (D) Overlay of structures from B and C. E) Results of pairwise comparison of structures in B and C showing RMSD (in Å) and TM values.

bioRxiv preprint doi: https://doi.org/10.1101/2024.08.22.609092; this version posted February 25, 2025. The copyright holder for this preprint (which was not certified by peer review) is the author/funder, who has granted bioRxiv a license to display the preprint in perpetuity. It is made available under aCC-BY 4.0 International license.

Fig S9. Paramyxovirus C protein structural modeling. (A) Protein maps of SeV C' and HPIV-1 C' showing region in SeV C' N-terminus sufficient for hSpt16^{SUMO} degradation (a.a. 12-34) aligned with corresponding region in HPIV-1 C'. NTE = N-terminal extension. Residues predicted to form alpha helical structures in the NTE are in red and purple for SeV C' and HPIV-1 C', respectively. (B) 6KP3 structure of SeV C' fragment (a.a. 99-204) bound to cellular Alix protein [62]. (C) Alphafold2-generated structure of full-length (a.a. 1-215) SeV C'. (D) Overlay of structures from B and C. (E) Results of pairwise comparison of structures in B and C showing RMSD (in Å) and TM values. (F) Alphafold2-generated structure of full-length (a.a. 1-219) HPIV-1 C'. (G) Overlay of structures from C and F. (H) Results of pairwise comparison of structures in B and C showing RMSD (in Å) and TM values.

Fig S10. Structural comparison of VSV M to paramyxovirus C proteins. (A) Overlay of Alphafold2-generated VSV M (Fig S8C), SeV C' (Fig S9C), and HPIV-1 C' (Fig S9F) structures. (B) Results of pairwise comparison of VSV M to either SeV C' or HPIV-1 C' structures showing RMSD (in Å) and TM values.

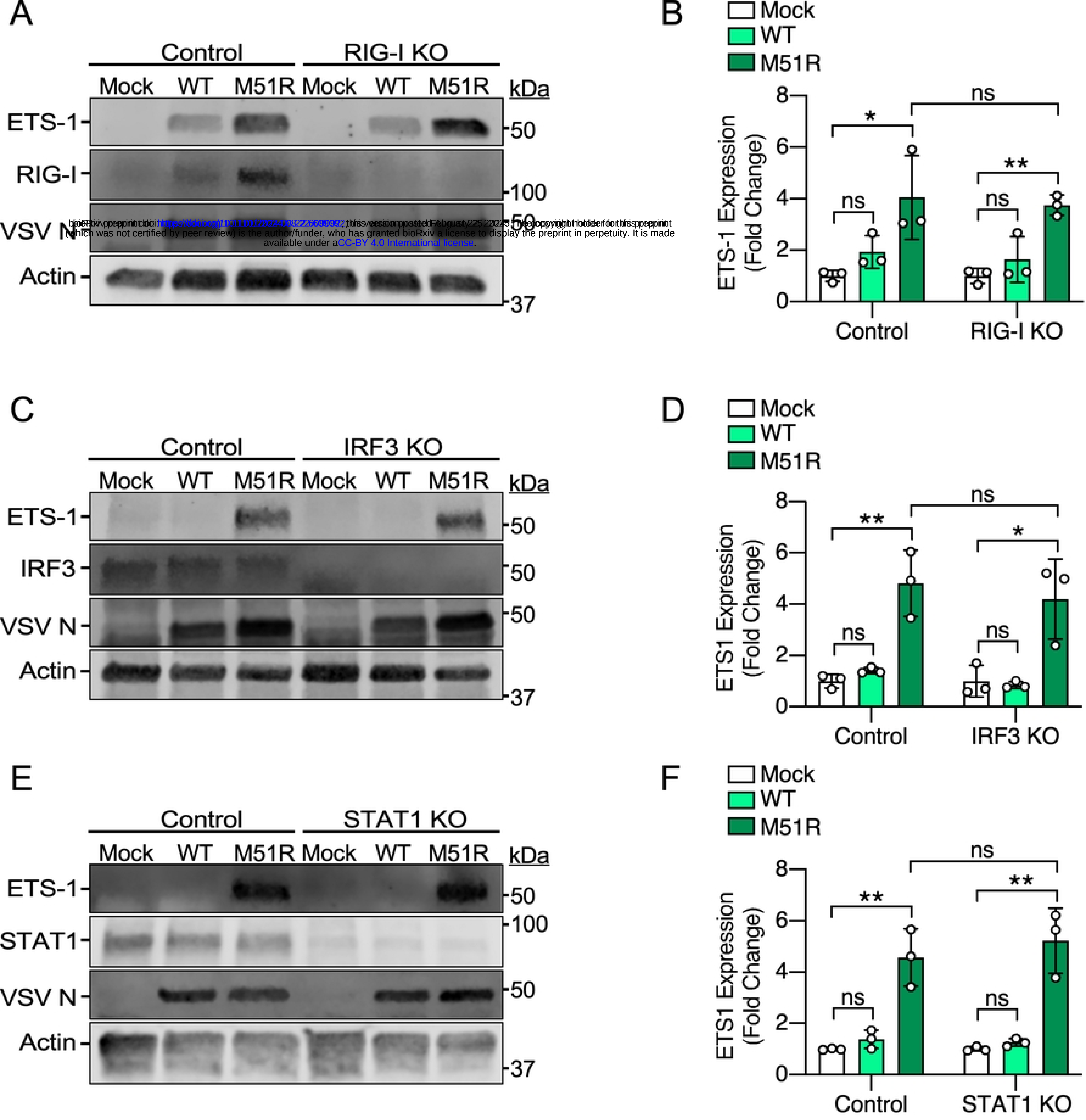

bioRxiv preprint doi: [illegible]; this version posted [illegible] 25, 2025. The copyright holder for this preprint (which was not certified by peer review) is the author/funder, who has granted bioRxiv a license to display the preprint in perpetuity. It is made available under aCC-BY 4.0 International license.

Figure S1

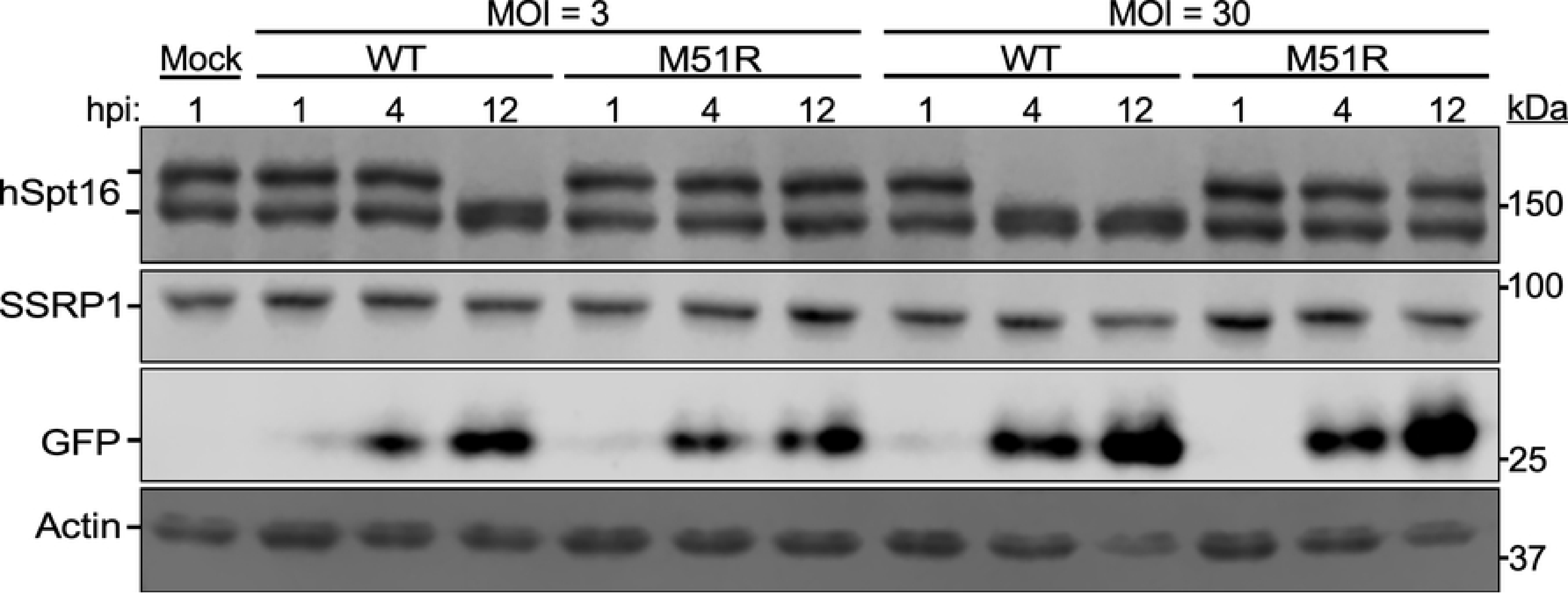
MOI = 3
MOI = 30
Mock
WT
M51R
WT
M51R
hpi:
1
1
4
12
1
4
12
1
4
12
1
4
12
kDa
hSpt16
SSRP1
GFP
Actin
150
100
25
37

Figure S2

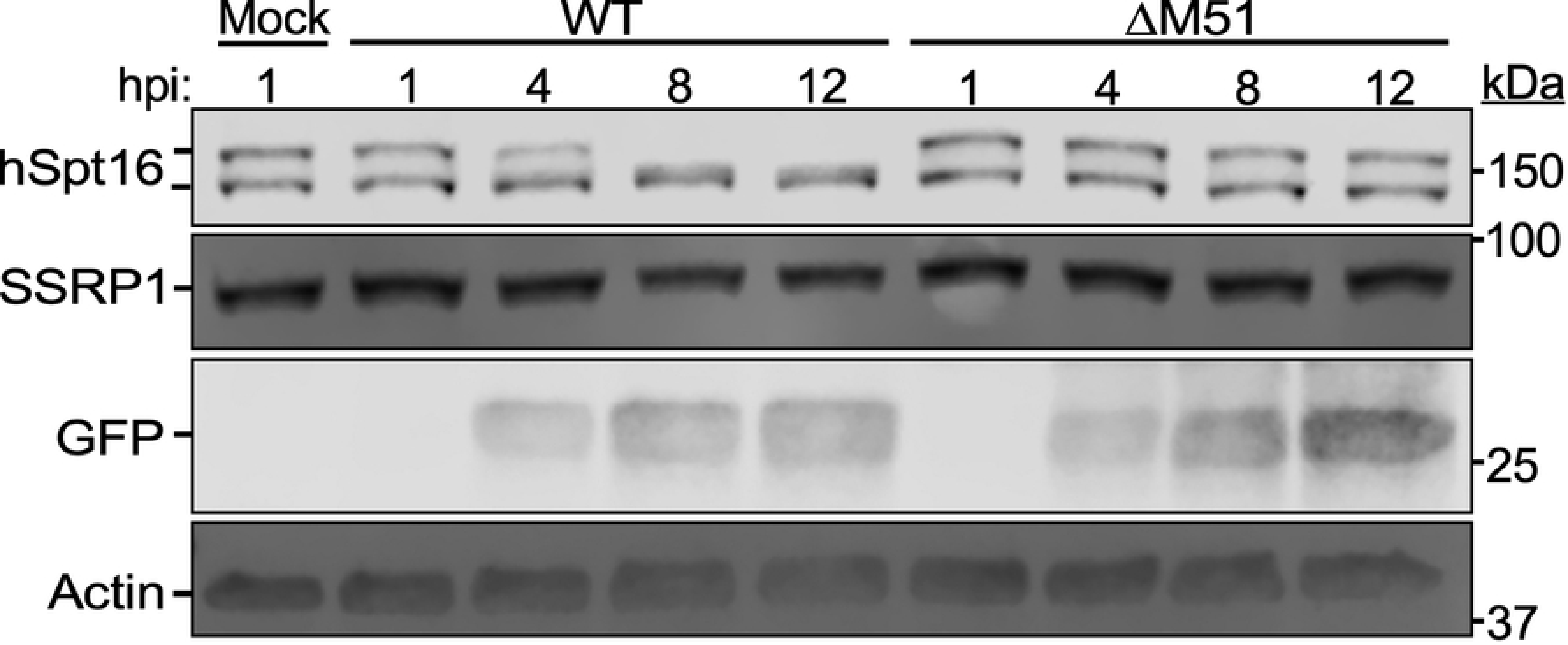
Mock
WT
ΔM51
hpi:
1
1
4
8
12
1
4
8
12
kDa
hSpt16
150
100
SSRP1
GFP
25
Actin
37
Figure S3

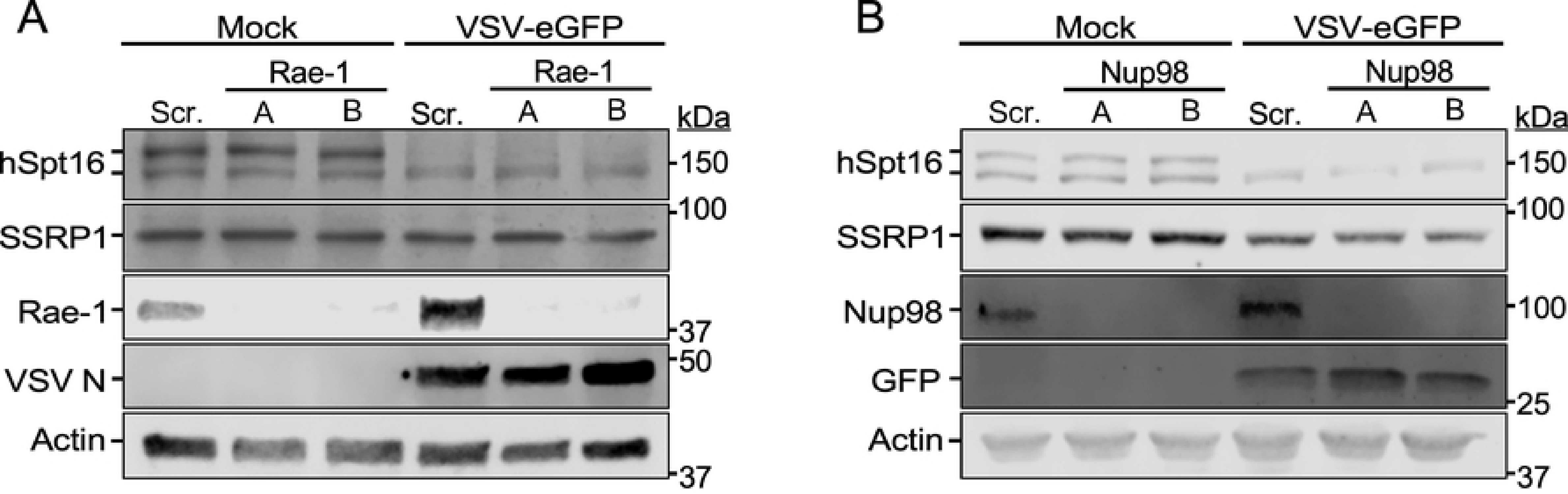
A
Mock
VSV-eGFP
Rae-1
Rae-1
Scr.
A
B
Scr.
A
B
kDa
hSpt16
150
100
SSRP1
Rae-1
37
50
VSV N
Actin
37
B
Mock
VSV-eGFP
Nup98
Nup98
Scr.
A
B
Scr.
A
B
kDa
hSpt16
150
100
SSRP1
Nup98
100
GFP
25
Actin
37

Figure S4

```
         a.a.
hSpt16   758 HDRDDLYAEQMEREMRHKLKTAFKNFIEKVEALTKEELEFEVPFRDLGFNGAPYRSTCLLQPTSSALVNATEWPPFVVTLDEVELI
LdSpt16  761 HDRDDLAAEQSERELRHKLKVAFKSFCERVETMTKQEVEFDTPFRELGFPGAPFRSTVLLQPTSGALVNLTEWPPFVIALEDVELV

hSpt16   844 HFERVQFHLKNFDMVIVYKDYSKKVTMINAIPVASLDPIKEWLNSCDLKY
LdSpt16  846 HFERVQFHLKNFDMVFVFKDYAKKVAMVNAVPMNMLDHVKEWLNSCDIRY
```

Figure S5

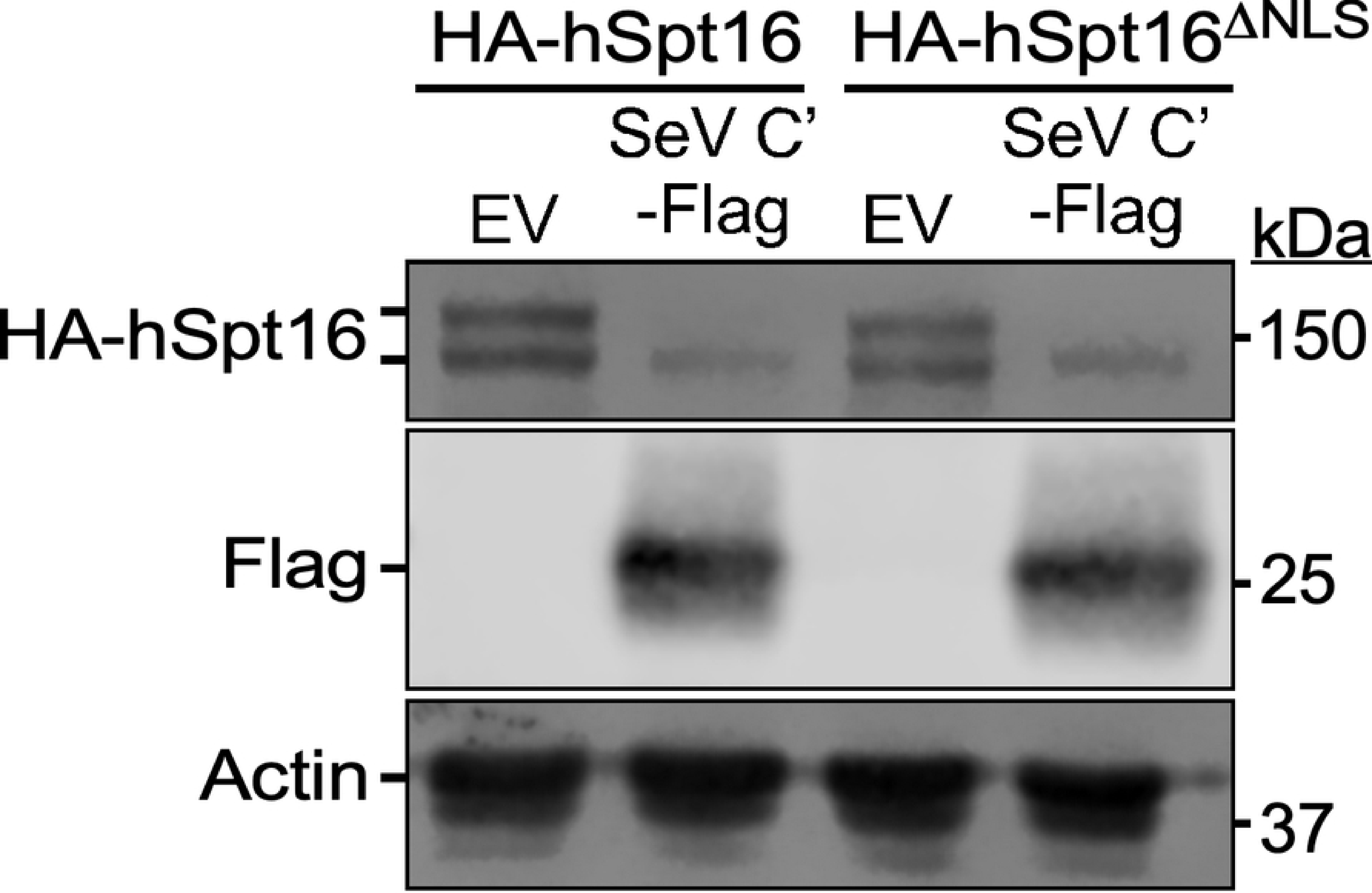
HA-hSpt16
HA-hSpt16ΔNLS
EV
SeV C' -Flag
EV
SeV C' -Flag
kDa
HA-hSpt16
150
Flag
25
Actin
37

Figure S6

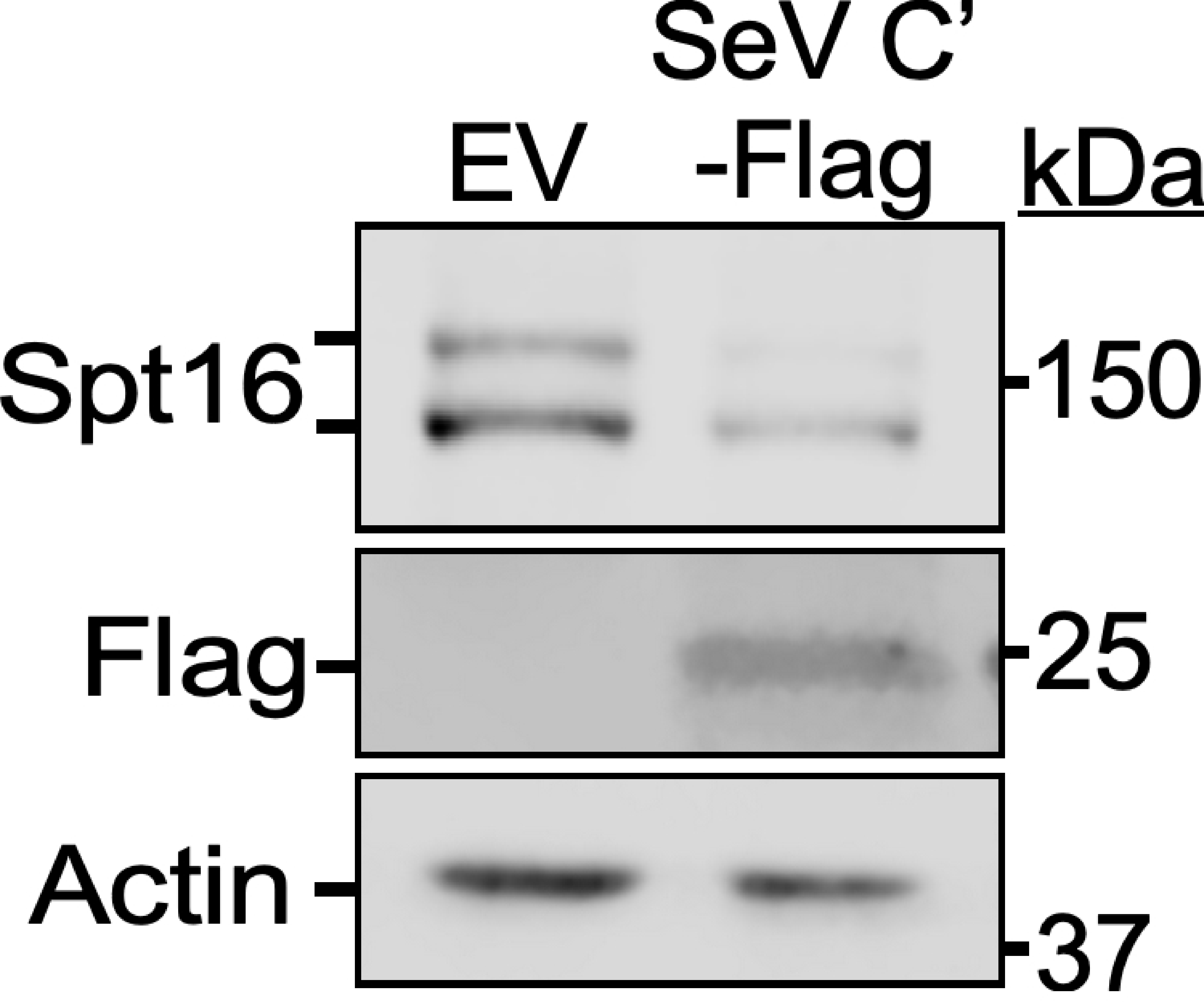

Figure S7

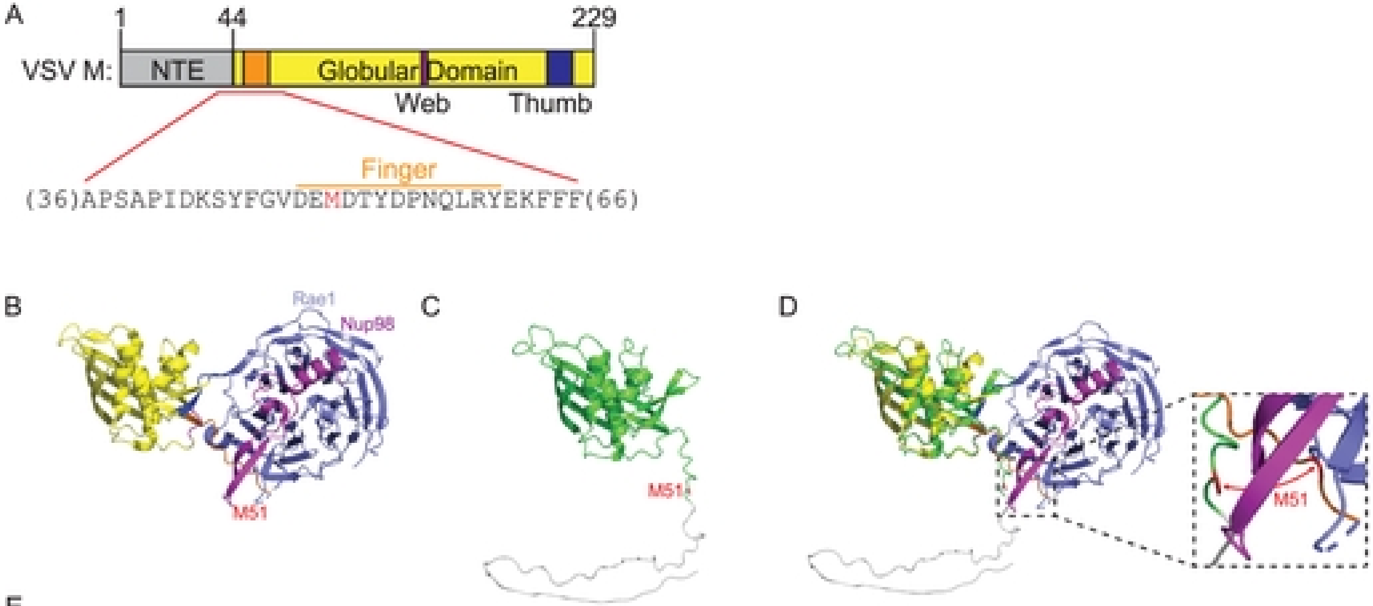

E

RCSB PDB Pairwise Structural Alignment

Entry	Length	RMSD	TM-Score	Identity	Aligned Residues
VSV M	1-229	0	1	100%	229/229
4OWR	42-229	1.82	0.93	97%	167/188

Figure S8

A

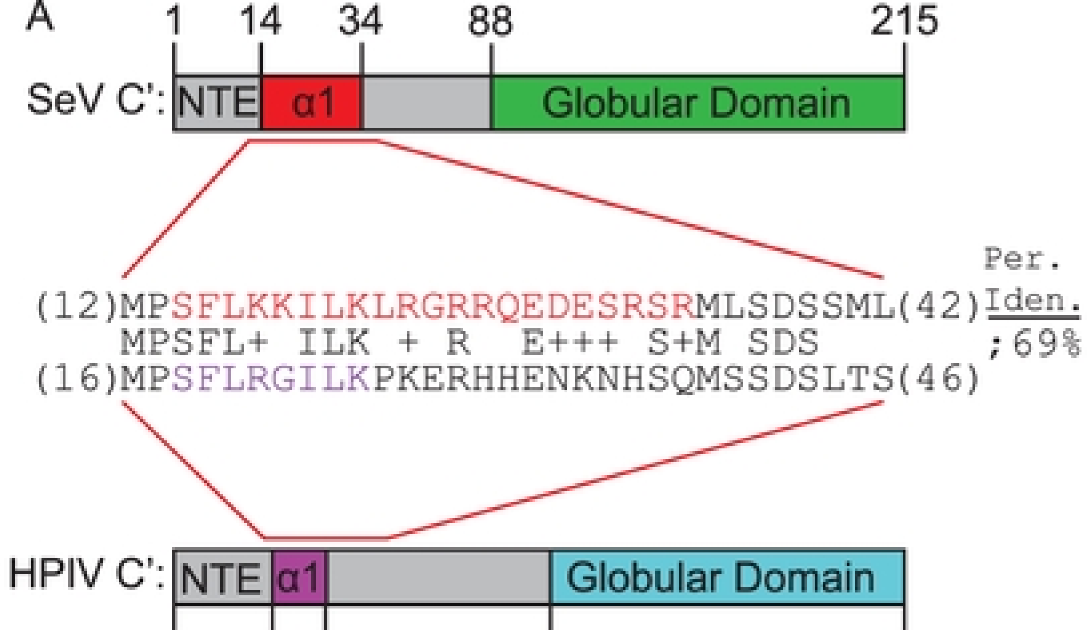

bioRxiv preprint doi: [illegible]; this version posted [illegible]. The copyright holder for this preprint (which was not certified by peer review) is the author/funder, who has granted bioRxiv a license to display the preprint in perpetuity. It is made available under aCC-BY 4.0 International license.

B C D

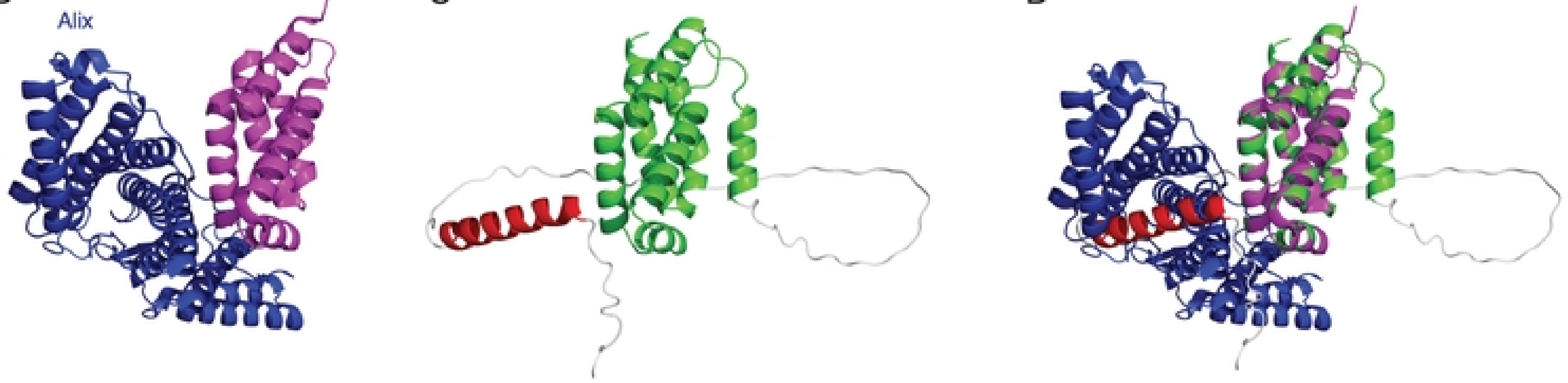

E

RCSB PDB Pairwise Structural Alignment

Entry	Length	RMSD	TM-Score	Identity	Aligned Residues
SeV C'	1-215	0	1	100%	215/215
6KP3	85-215	1.22	0.92	92%	109/119

F G

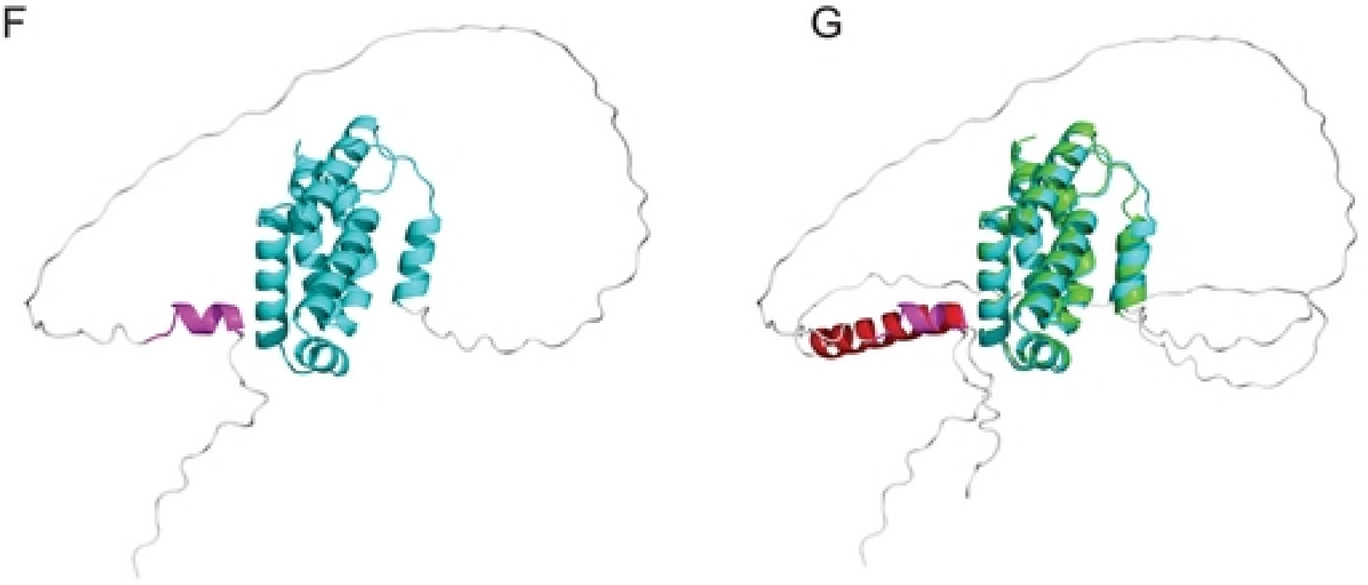

H

RCSB PDB Pairwise Structural Alignment

Entry	Length	RMSD	TM-Score	Identity	Aligned Residues
SeV C'	1-215	0	1	100%	215/215
HPIV-1 C'	1-219	2.57	0.66	77%	144/219

Figure S9

A

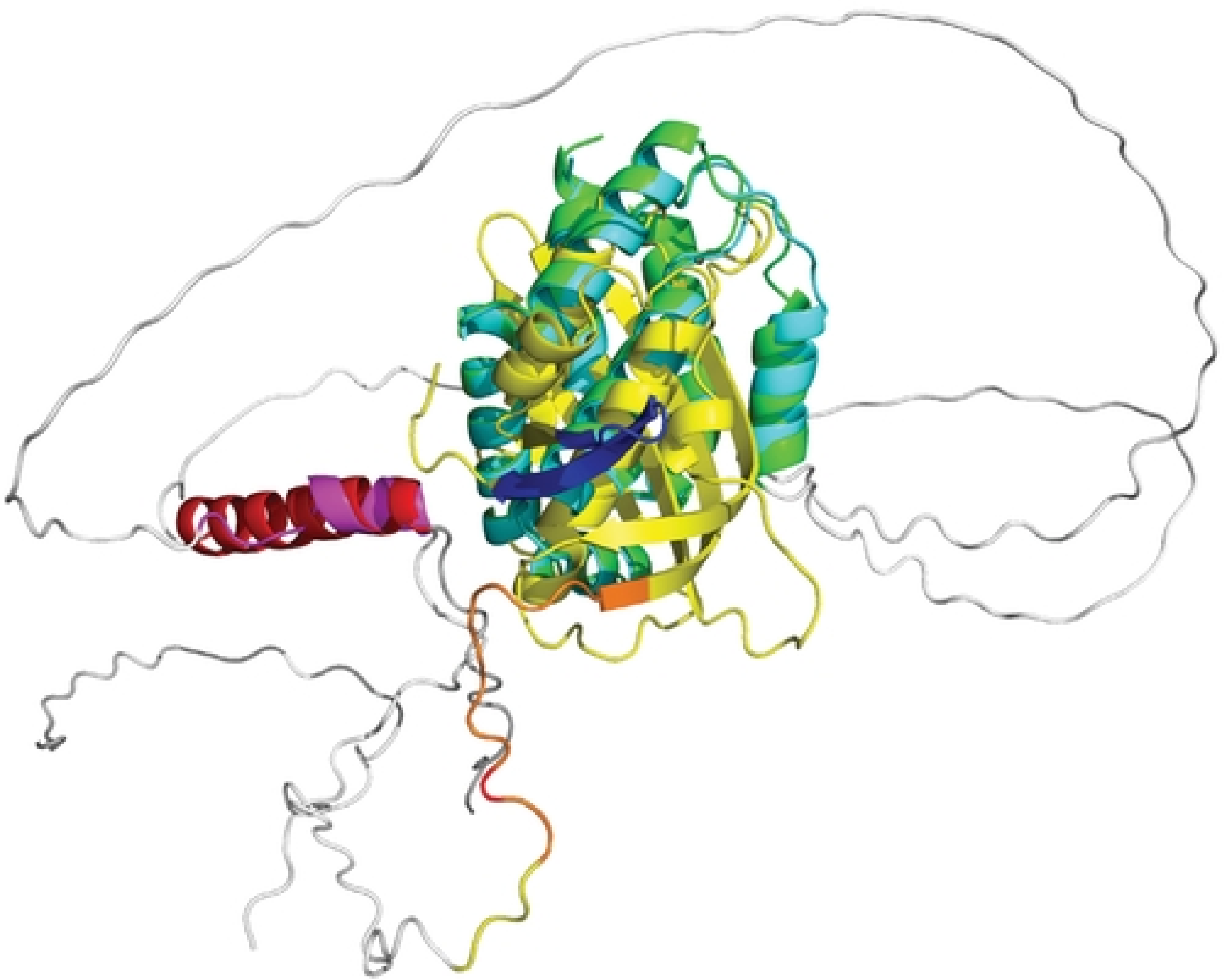

B

RCSB PDB Pairwise Structural Alignment

Entry	RMSD	TM-Score	Identity	Aligned Residues
VSV M	0	1	100%	229/229
SeV C’	5.52	0.22	7%	46/216
HPIV-1 C’	6.45	0.21	5%	43/219

Figure S10
